# No Need to Discriminate? Reproductive Diploid Males in a Parasitoid with Complementary Sex Determination

**DOI:** 10.1371/journal.pone.0006024

**Published:** 2009-06-24

**Authors:** Jan Elias, Dominique Mazzi, Silvia Dorn

**Affiliations:** Institute of Plant Sciences, Applied Entomology, Swiss Federal Institute of Technology Zurich (ETH Zurich), Zurich, Switzerland; Aarhus University, Denmark

## Abstract

Diploid males in hymenopterans are generally either inviable or sterile, thus imposing a severe genetic load on populations. In species with the widespread single locus complementary sex determination (sl-CSD), sex depends on the genotype at one single locus with multiple alleles. Haploid (hemizygous) individuals are always males. Diploid individuals develop into females when heterozygous and into males when homozygous at the sex determining locus. Our comparison of the mating and reproductive success of haploid and diploid males revealed that diploid males of the braconid parasitoid *Cotesia glomerata* sire viable and fertile diploid daughters. Females mated to diploid males, however, produced fewer daughters than females mated to haploid males. Nevertheless, females did not discriminate against diploid males as mating partners. Diploid males initiated courtship display sooner than haploid males and were larger in body size. Although in most species so far examined diploid males were recognized as genetic dead ends, we present a second example of a species with sl-CSD and commonly occurring functionally reproductive diploid males. Our study suggests that functionally reproductive diploid males might not be as rare as hitherto assumed. We argue that the frequent occurrence of inbreeding in combination with imperfect behavioural adaptations towards its avoidance promote the evolution of diploid male fertility.

## Introduction

Most of the over 200,000 species in the insect order Hymenoptera, which includes wasps, bees and ants, have an arrhenotokous haplodiploid life cycle. Under arrhenotoky, diploid females develop from fertilized eggs and haploid males from unfertilized eggs [1]–[3]. Nevertheless, under single locus complementary sex determination (sl-CSD), diploid males can occur. Out of several proposed mechanisms of sex determination in the insect order Hymenoptera, sl-CSD is the best understood and supported [4], [5]. Under sl-CSD, the sex of an individual is determined by one single locus with multiple alleles [2], [6], [7]. Diploid individuals that are heterozygous at the sex locus develop into females, whereas hemizygous (haploid) and homozygous diploid individuals develop into males [6], [8]. sl-CSD has been demonstrated in more than 60 species within the Hymenoptera [9]–[12], among them the gregarious endoparasitoid wasp *Cotesia glomerata* (Hymenoptera: Braconidae) [5], [13].

In species with sl-CSD, random mating results in the occasional occurrence of matched matings, matings between individuals carrying an identical allele at the sex determining locus [14]. Within a randomly mating population with k sex alleles and in which diploid males are viable, the probability of a matched mating is 2/k. Under matched matings, half of the diploids develop into males. Thus, in a randomly mating population, the probability of a diploid developing into a male is 1/k [4], [6]. However, in the event of a mating between siblings of unmatched parents, the probability of a matched mating is always 0.5, independently of k. Furthermore, in the case of matched parents, all sib-matings will be matched. Thus, when siblings mate, the frequency of matched matings equals or exceeds 0.5 [4]–[6]. Because half of the offspring resulting from matched matings develop into diploid males, their frequency under sib-mating is at least 0.25.

In general, diploid males are considered a genetic load for a population due to their low viability [2], [5], [15], inability to mate properly [16], sterility [17]. Furthermore, some species were found to produce fertile diploid males, which on their part produced sterile triploid offspring [18]–[20]. Therefore, diploid males represent a cost to their parents, as they perform poorly in traits associated with fitness, and represent a cost to females mating with them, as they will produce sterile, if any, progeny [6], [21]. Thus, species with sl-CSD are expected to have evolved inbreeding avoidance mechanisms [6].

One such mechanism is the egg-laying pattern of the turnip sawfly *Athalia rosae* (Hymenoptera: Tenthredinidae), where fertilized eggs are laid early in a female's life and unfertilized eggs later on, thus leading to a temporal segregation of opposite sex-kin [22]. Spatial segregation of opposite-sex kin may similarly contribute to avoid inbreeding. In *C. glomerata*, over 50% of females and 30% of males leave their natal patch before mating [23]. In the parasitic, gregarious wasp *Bracon hebetor* (Hymenoptera: Braconidae), females and males are unreceptive to mating during the first two hours after emergence, and indeed most females depart the site of emergence unmated [24], [25]. Furthermore, olfactory signals have been shown to matter in the discrimination against related partners [26]. Additional potential mechanisms to reduce the diploid male load are discussed in [12].

Contrary to expectation, some species engage in inbred matings despite having sl-CSD. One such species is the solitary parasitoid wasp *Euodynerus foraminatus* (Hymenoptera: Vespidae) [4], [27]. It has recently been shown that diploid males in *E. foraminatus* have normal fertility and father fertile diploid daughters at a rate comparable to that of haploid males. The daughters of diploid males produced viable, fertile daughters of their own [21]. So far, this is the only species with documented normal fertility of diploid males and has not yet been incorporated into models predicting implications of diploid male production at the population level [3]. A model simulating the role of diploid males that impose a genetic load to populations was lately proposed by Zayed and Packer [28]. Their results indicated that diploid male production can initiate a vortex that makes small, isolated populations of species with sl-CSD particularly prone to extinction. Given the potential detrimental implications of sl-CSD at the population level, it is of utter importance to understand the cost of diploid male production and diploid male function in general.

Zhou and co-workers recently provided first solid evidence for the occurrence of sl-CSD in the genus *Cotesia*, and of viable diploid males in the gregarious endoparasitoid wasp *C. glomerata* [5], [13]. In an earlier study, a sib-mating ratio as high as 60% was estimated for *C. glomerata* [29], suggesting systematic inbreeding to be typical for this species. Species with high levels of inbreeding are expected to suffer from high costs of diploid male production. Thus, it is important to advance the understanding of diploid male function in *C. glomerata* by addressing the question of whether diploid males are sexually competent and fertile, and whether their putative daughters are in turn fertile.

## Results

### Mating success of haploid and diploid males

Ploidy analysis by flow cytometry revealed 51 haploid males and 26 diploid males throughout our 13 replicates. Within a replicate, one to four diploid males were present. One male whose ploidy could not be determined was omitted from the analyses. Mating behaviour differed between haploid and diploid males (Table 1). We found a significant effect of ploidy on the onset of courtship behaviour. Diploid males started courting females sooner than haploid males. Even though copulation latency of diploid males was almost 45 seconds shorter than that of haploid males, the difference fell short of statistical significance. The total number of attempts to mount a female did not differ significantly between haploid and diploid males. Mating success of diploid and of haploid males was comparable in our experiment (Table 2). We found no significant effect of ploidy on either observed mating success (P = 0.54, R^2^ = 0.51) or on mating success as defined by the occurrence of daughters (P = 0.20, R^2^ = 0.45). Diploid males were significantly larger than haploid males (Table 1).

**Table 1:** Mating behaviour and a proxy of body size of haploid and diploid males.

	Courtship latency [sec]	Copulation latency [sec]	Mating attempts	Tibia length [mm]
Haploid males	155±38	857±63	2.1±0.4	0.89±0.01
Diploid males	151±52	812±92	1.7±0.4	0.90±0.01
P	0.02	0.06	0.19	0.01

Mean values and standard error calculated over the pooled dataset are given, whereas P-values refer to within-replicate comparisons of haploid and diploid males.

**Table 2:** Mating success of diploid and haploid males as observed in behavioural trials and as defined by the occurrence of daughters.

Ploidy	Mating success	
	Observed	Occurrence of daughters
	Successful/Total	Successful/Total
Haploid	21/51	31/51
Diploid	13/26	16/26
Total	34/77	47/77
P	0.54	0.20

### Reproductive success of haploid and diploid males

Little difference was noted in reproductive success between haploid and diploid males (Table 3). Each female mated to either a diploid or a haploid male parasitized 20 host larvae. Our analysis showed no significant effect of ploidy on the total number of cocoons egressed from the host larvae. The comparison of the offspring of diploid and haploid males revealed no significant difference with regard to the production of sons, but diploid males produced significantly fewer daughters than haploid males did. The difference in the total number of cocoons per cluster produced by females mated to diploid males and by females mated to haploid males fell just short of significance, indicating a trend towards females mated to diploid males to lay more eggs. The sex-ratio of broods fathered by haploid and diploid males was not significantly different. The proportion of emerged adult offspring did not differ significantly between haploid and diploid fathers either.

**Table 3:** Reproductive success of haploid and diploid males.

	Egressed cocoons	Sons	Daughters	Cocoons/cluster	Sex-ratio	Hatching success
Haploid males	7.2±0.8	10.4±1.5	12.4±1.7	29.2±2.6	0.48±0.05	0.61±0.05
Diploid males	6.7±0.8	15.0±2.3	9.9±2.3	29.7±2.9	0.62±0.07	0.70±0.06
P	0.80	0.37	0.02	0.07	0.41	0.13

Mean values and standard error calculated over the pooled dataset are given, whereas P-values refer to within-replicate comparisons of haploid and diploid males.

The 16 randomly selected daughters of four diploid males were all diploid. Furthermore, five out of 15 daughters of three diploid males and two out of 15 daughters of three haploid males produced daughters of their own.

## Discussion

Our data show that diploid males in *C. glomerata* are as competitive as haploid males in obtaining matings and father viable and fully fertile diploid daughters. Thus, diploid males in *C. glomerata* are sexually competent and fertile. Although mating success was similar under our experimental conditions, we identified significant differences between haploid and diploid males with respect to their initiation of courtship behaviour, body size, and reproductive output. Diploid males initiated courtship display earlier, were larger and sired fewer daughters than haploid males.

Previous studies of hymenopteran species with sl-CSD mostly recognized diploid male production to be a genetic dead end (see Table 4). However, widespread generalizations were recently questioned when diploid male fertility was reported in the solitary hunting wasp *Euodynerus foraminatus* [21]. The question arises as to how diploid males may pass on a haploid set of chromosomes to their daughters. In *C. glomerata* [5] and several other species with sl-CSD [18]–[20], diploid males produce diploid sperm. This appears to be the result of a spermatogenesis aborted during metaphase in the first reductional division (meiosis I), as it occurs in haploid males, resulting in two rather than the typical four spermatids [30], [31]. Our finding that diploid males are reproductive suggests that one out of their two chromosome sets is eliminated during the fertilization process, resulting in the production of a diploid zygote [21]. Alternatively, a modification of paternal genome elimination (PGE) as found in the parasitoid wasps *Nasonia vitripennis* (Hymenoptera: Pteromalidae) [32], [33] and *Trichogramma kaykai* (Hymenoptera: Trichogrammatidae) [34], [35] might enable diploid males to produce diploid daughters. During mitosis of a triploid zygote, one set of paternal chromosomes could be eliminated and the resulting diploid zygote would eventually develop into a daughter. As a result, females sired by diploid males obtain two sets of chromosomes. We exclude thelytoky (parthenogenetical development of females from unfertilised eggs) as a possible explanation for the observed daughters of females mated to diploid males. Under thelytoky unfertilised females produce only daughters [3]. In our experiment, however, females mated to diploid males produced a number of sons comparable to that of females mated to haploid males. A co-occurrence of thelytoky and arrhenotoky is so far known in two species, the ichneumonid *Venturia canescens* [36] and a South African strain of the Cape honey bee, *Apis mellifera capensis* [37], [38]. In *V. canescens*, individuals of the two reproductive modes are spatially separated, whereas in *A. mellifera capensis*, the separation of reproductive modes is across the caste boundary, with workers being thelytokous and queens being typically arrhenotokous. However, it appears that queens are arrhenotokous when fertilised but may choose between arrhenotoky and thelytoky when laying unfertilised eggs [39]. If there was any form of thelytoky occurring in our system, then, occasionally, we would obtain daughters of virgin females. We never detected a single female among the progeny of hundreds of virgin mothers, ruling out co-occurrence of thelytoky and arrhenotoky. Consistently, all of our findings confirm the repeatedly described arrhenotokous life cycle of the species [reviewed in 40].

**Table 4:** Chronological overview of earlier studies of the fertility of diploid males in other species.

Species (Family)	Diploid males	Daughters of diploid males		Lifestyle	Reference
		Ploidy	Fertility		
*Habrobracon hebetor* (Braconidae)	sterile	–	–	gregarious ectoparasitoid	[2], [15]
*Apis mellifera* (Apidae)	cannibalized by workers	–	–	social	[64]
*Neodiprion nigroscutum* (Diprionidae)	fertile (<1% mating success)	3N	sterile	gregarious	[16]
*Solenopsis invicta* (Formicidae)	sterile and few fertile	3N	non-reproductive	social	[65], [66]
*Athalia rosae* (Tenthredinidae)	fertile	3N	sterile	gregarious	[19]
*Diadromus pulchellus* (Ichneumonidae)	sterile and few fertile	2N	fertile	solitary endoparasitoid	[17]
*Lasius sakagamii* (Formicidae)	possibly fertile	3N	partially fertile	social	[18]
*Bombus terrestris* (Apidae)	fertile	3N	sterile	social	[67]
*Euodynerus foraminatus* (Vespidae)	fertile	2N	fertile	solitary	[21]
*Polistes dominulus* (Vespidae)	fertile	3N	probably sterile	social	[68]
*Cotesia vestalis* (Braconidae)	fertile	3N	sterile	solitary endoparasitoid	[20]

The reproductive potential of diploid males in a species with sl-CSD was first described by El Agoze et al. [17]. Diploid males in the solitary parasitoid wasp *Diadromus pulchellus* (Hymenoptera: Ichneumonidae) sired diploid daughters, albeit as few as 1% of the total offspring. Despite low productivity, the occurrence of diploid male fertility in species with sl-CSD may be a crucial adaptation for the survival of populations. Inbreeding, as it occurs in *C. glomerata* and *E. foraminatus* [27], [29], and the loss of allelic diversity at the sex locus can substantially increase diploid male production [13], [20]. In small isolated populations, extrinsic natural and anthropogenic factors (e.g. habitat loss and fragmentation, use of pesticides) may lead to a decrease in population size, resulting in frequent inbreeding, increased diploid male production and ultimately population extinction [28]. Consequently, hymenopterans with sl-CSD are expected to evolve behavioural mechanisms that promote outbreeding, as is the case in the honey bee *Apis mellifera* [14], [41] or the braconid wasp *Bracon hebetor* [24], [25]. In *C. glomerata*, the frequent occurrence of superparasitism [42], [43] may create the opportunity for matings between non-siblings emerging from the same host, thus promoting outbreeding. In nature, sex allele diversity is typically high and maintained by frequency-dependent selection favouring rare alleles, thus, diploid male frequencies are generally low [44], [45]. Nevertheless, if a species fails to develop a behavioural mechanism to avoid inbreeding and the overall inbreeding frequency increases due to habitat fragmentation or other extrinsic factors, small populations might face the risk of extinction. Selection for fertile diploid males could follow and rescue a population from the far-reaching deleterious consequences of diploid male production [21]. Thus, populations might persist, if only a few diploid males reproduce. *D. pulchellus* appears to be a species with variability in the reproductive potential of diploid males, whereas *E. foraminatus* already has fully fertile diploid males and might have evolved out of a state comparable to present *D. pulchellus*. Diploid males in *C. glomerata* produce fewer daughters than haploid males, possibly indicating that the fertilization process works less reliably than that of haploid males. Hence, we tentatively speculate that *C. glomerata* is a species where diploid male fertility is still progressing towards a comparable efficiency of that of haploid males.

In *C. glomerata*, sexes emerge asynchronously, and males often stay close to the natal patch waiting for the later-emerging females [29]. Upon emergence of the females, males from the same cluster as well as those dispersed from other clusters compete for mating opportunities. Gu and Dorn [23] observed males fighting each other over access to females at their natal patch and described the occurrence of partial local mate competition in *C. glomerata*. Large males often have greater fighting ability than smaller males [46], [47]. Diploid males in *C. glomerata* were larger in size than haploid males, initiated courtship display sooner and tended to require less time to obtain a mating. Thus, they may enjoy a competitive advantage over haploid males.

Nevertheless, females mated to diploid males produced fewer daughters than females mated to haploid males. In order to obtain comparable reproductive success, diploid males would need to mate more frequently than haploid males. They might achieve greater mating success by defeating haploid males in fights, and by courting and mounting females more eagerly. Thus, although we found diploid males to produce fewer daughters when mated to a single female, they may offset the deficit through increased mating activity when females are abundant.

Although at present we ignore how common fertile diploid males are in natural populations, they present new challenges to populations. Females adjust the sex-ratio of their offspring by controlling sperm release to an egg during oviposition [12]. Inbreeding increases the occurrence of matched matings and, therefore, the occurrence of diploid males among a female's progeny. Thus, mating with a close kin leads to a decreased control over the sex-ratio of the offspring. To circumvent the loss of control, behavioural mechanisms have evolved. For example, natal dispersal may lower the chances of sibling encounters in *C. glomerata* [23]. Both sexes in this species are strong flyers [48] and females use host-induced plant volatiles to locate suitable oviposition sites [49], promoting gene flow and genetic exchange. Queens of the honey bee *Apis mellifera* are polyandrous [14], [41], thus bet-hedging against sperm matched to their own sex locus allele. In populations with five or fewer sex determining alleles, multiple matings are favoured because females that mate more than once may have higher than average fitness [50]. Polyandry might be a suitable way out for inbreeding *C. glomerata* females, provided that the odds that their eggs are fertilised with a compatible sperm (i.e. unmatched at the sex locus) are improved and sex determining allele diversity is low within populations [50]–[54]. Females mated to haploid males have half of their genome represented in haploid and diploid offspring. When their haploid sons reproduce, the sons pass on all of their genome to their daughters. Thus, the mothers of the haploid sons are represented in their granddaughters or diploid grandsons with half of their genome as well. The production of diploid sons rather than of haploid sons leads to an under-representation of maternal genetic material in grandchildren. Diploid males pass on genetic material inherited from either their mother or their father to their daughters. Under these circumstances, haploid males profit from sib-matings, because they are genetically represented in diploid male offspring and also in successive generations. Thus, diploid males are more valuable to fathers but less valuable to mothers and their occurrence might fuel sexual conflict over the allocation of parental resources.

In contrast to the solitary wasp *E. foraminatus*, *C. glomerata* is gregarious. Our study shows that the finding of functionally reproductive diploid males in *E. foraminatus* [21] is not a unique exception, and not necessarily restricted to solitary wasps. Under ideal conditions (e.g. continuous habitats and sufficient gene flow) we would expect more seldom kin encounters in solitary than in gregarious species, where kin develop on or in the same host. Because the ability to discriminate against siblings appears to be better developed in species in which kin meet each other more frequently as adults [55], [56], we would expect gregarious species to adopt behavioural strategies to reduce the occurrence of inbreeding, rather than to evolve physiological modifications enabling diploid male fertility. In *E. foraminatus*, female wasps lay their eggs in a linear sequence of cells, each containing one egg provisioned with paralysed caterpillars [27]. Thus, although strictly a solitary species, the close proximity of kin within nests suggests that siblings meet each other as adults at their nesting site. Consequently, we would have predicted the evolution of behavioural adaptations towards inbreeding avoidance rather than diploid male fertility both in *E. foraminatus* and in *C. glomerata*. Yet, both species appear to engage in frequent systematic inbreeding [27], [29]. *C. glomerata* is less related to *E. foraminatus* than to the solitary parasitoid wasp *Cotesia vestalis*. Yet, diploid males in *C. vestalis* were able to mate, but produced triploid progeny [20]. Recently, experimental support was provided for multiple locus complementary sex determination (ml-CSD) in *C. vestalis* [57], [58], suggesting that ml-CSD is another evolutionary solution to the diploid male load. Furthermore, two species of the same genus, *Cotesia flavipes* and *Cotesia sesamiae*, do not have CSD [59] and hence circumvent the genetic load by avoiding diploid male production altogether. It thus appears that the evolution of diploid male fertility is neither dependent on a solitary or gregarious lifestyle nor on taxon, but rather has evolved repeatedly and independently.

Although diploid male fertility is probably rare among hymenopterans with sl-CSD, we agree with the cautionary note by Cowan and Stahlhut [21] urging against generalizations about diploid male function in the Hymenoptera. Furthermore, we emphasize the need to include the scenario that diploid males sire diploid daughters in theoretical models of population dynamics to better understand its implications for the conservation of hymenopteran species in the face of environmental change.

## Materials and Methods

### Insect rearing

Adult *C. glomerata* wasps were reared in insect cages (30×30×30 cm) kept at 15°C, 70% relative humidity (r.h.) under a light-dark regime of 16h∶8h. They were fed honey and water ad libitum, refreshed twice a week. To maintain the colony, the herbivorous host *Pieris brassicae* (Lepidoptera: Pieridae) was used. Larvae of *P. brassicae* were reared on Brussels sprout plants (*Brassica oleracea* var. *gemmifera*) in an insectary at 21±1°C, 60% r.h., 16L:8D. Twice a week, up to 30 second instar *P. brassicae* larvae were introduced into the insect cages containing *C. glomerata* to be parasitized and removed after ten minutes to avoid superparasitism [43]. The parasitized host larvae were reared in the insectary at 21±1°C, 60% r.h., 16L:8D. After egression (of mature parasitoid larvae from the host [60]) and subsequent pupation, cocoon clusters of *C. glomerata* were collected and kept at 15°C, 70% r.h., 16L:8D. Upon emergence (of adult parasitoid wasps [60]) wasps were placed in an insect cage, and the cycle started anew.

### Mating success of haploid and diploid males

In order to compare mating success of diploid and haploid males, we used a culture established over the summer of 2006 and a newer one established in 2007, both derived from cocoon clusters sampled in a large cabbage-growing area near Unter-Stammheim, Zurich, Switzerland (47°38' N, 8°46' E). We assumed that the occurrence of matched matings and thus the chances of picking clusters including both haploid and diploid males as required for the experimental set-up would increase, the longer a culture is maintained in the laboratory [13]. Females to be used in behavioural trials were taken from the culture established in 2007 in order to minimize the inbreeding effects ensuing from prolonged laboratory rearing, whereas males were taken from the culture established in 2006.

The behavioural trials ran between June 2007 and February 2008. For each replicate, we used one cocoon cluster from the 2007 culture and one cocoon cluster from the 2006 culture. Single cocoons were kept individually in 1.5 ml vials. We randomly chose six virgin females (i.e. sisters or half-sisters) from the cocoon cluster out of the 2007 culture and six virgin males (i.e. sons of the same mother) from the cocoon cluster out of the 2006 culture, to minimize within-replicate variation. Each male was tested for mating success once with one randomly allocated female. All males and females were used only once. Thus, one replicate consisted of six mating trials. Only 2–5 day old males and females were used. A male and a female were placed in a Petri dish (∅ 5.5 cm) divided into two sections by gauze fixed with PVC glue. Pairs were placed in one section. To promote mating with a standardized stimulus signalling the presence of hosts on plants [61], in the other section 50 ml of a hexane extract consisting of a plant-host complex were applied to a Whatman filter paper disc (∅ 2 cm, Whatman International Ltd., Maidstone, U.K.). The hexane extract from the plant-host complex consisted of host frass, host exuviae and host infested cabbage leaves. 5 g frass, 0.2 g exuviae and 1.5 g damaged cabbage leaves were soaked in 100 ml 99.5% hexane at room temperature for 30 min and then kept at −60°C for 36 h. The supernatant was stored in 0.5 ml aliquots at −80°C. The arena was illuminated from above by a jitter-free fluorescent tube (Osram W58/L12 daylight LUMILUX de Luxe, Osram GmbH, Munich, Germany) with a light intensity at the arena location of approximately 4,000 Lux, at a temperature of 25±2°C and r.h. of 40%. Males and females were acclimatized to the experimental conditions for at least 2 h prior to the start of experiments.

Trials were watched continuously for 20 min or until mating occurred. The occurrence of mating was defined as the male staying in contact with the female for at least 30 sec. Furthermore, courtship latency (defined as the time until the start of the characteristic male wing-fanning display), copulation latency (defined as the time until the start of copulation) and the number of mating attempts by the male were recorded. Thirteen replicates were performed (i.e. thirteen replicates consisting of six mating trials each, totaling 78 mating trials).

### Reproductive success of haploid and diploid males

After the behavioural trials, pairs were placed for another 24 hours in a 1.5 ml vial at 15°C, 70% r.h. under a light-dark regime of 16 h∶8 h to allow mating had it not yet occurred. Thereafter, all males were stored at −80°C for later measurements of body size and ploidy analyses. As a proxy of body size, we measured the right hind tibia length at 50× magnification under a Wild M5A stereomicroscope (former Wild Heerbrugg AG, Switzerland) equipped with an ocular micrometer with a scale etched with divisions of 0.016 mm. Tibia length is a reliable correlate of body size (own unpublished data). Females were allowed to parasitize 20 *P. brassicae* larvae each for assessment of reproductive success. Parasitized host larvae were reared as described above. The number of egressed cocoon clusters was noted and single cocoons were isolated into 1.5 ml vials in order to determine hatching success (i.e. the proportion of hatched eggs), brood size (i.e. the number of emerged adult offspring) and sex-ratio (i.e. the proportion of male offspring). Production of daughters was regarded as proof of successful insemination. Since daughters were obtained from the progeny of diploid males, we randomly selected 15 daughters of three diploid and three haploid fathers (five daughters per male) and paired them with unrelated haploid males to check whether they were fertile.

### Flow cytometric analysis of ploidy

The ploidy of males used in the mating trials and of the progeny of mated diploid males was determined using a Partec Ploidy Analyzer PA-II (Partec GmbH, Münster, Germany), equipped with an HBO mercury arc lamp for ultraviolet excitation. Thawed *C. glomerata* samples were crushed using the tip of a pipette and 100 µl of CyStain® UV Ploidy staining solution (Partec GmbH, Münster, Germany) containing the DNA-specific fluorochrome DAPI (4′6-diamidino-2-phenylindole) were added. After an incubation time of 55 seconds, further 300 µl of staining solution were added. Thereafter, the solution was filtered through a 50 µm CellTric® filter (Partec GmbH, Münster, Germany) and another 600 µl of staining solution were added through the filter. After briefly vortexing, 300 µl of solution were transferred to a small insert together with 600 µl ddH_2_O. The diluted solution was used for analysis.

We analyzed the ploidy of all males used in the behavioural experiment, and of 16 randomly selected females sired by four diploid males. A (necessarily diploid) female and a haploid male (a son of a virgin female) were used as calibration references.

### Statistical analyses

Data were analyzed using the R software [62] together with the lattice package [63].

To test for differences in mating success between diploid and haploid males, we applied a logistic regression model and performed a chi squared likelihood ratio test. We tested for both mating success as observed in behavioural experiments and mating success as defined by the occurrence of daughters. All other variables were tested performing an analysis of variance (ANOVA) with replicate and ploidy as fixed effects. Where required, variables were log- or square root- transformed to better approach normal distribution of residuals.

**Figure 1:**
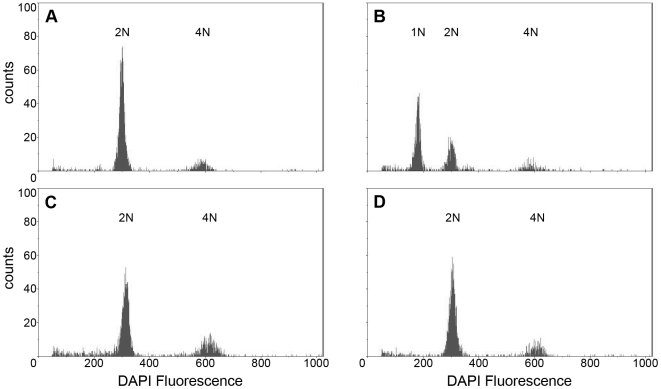
Flow cytometric histograms of DAPI stained DNA. A representative diploid female (A), a haploid male (B), a diploid male (C) and a daughter of the diploid male (D) are illustrated. Diploid females and sons of virgin females, necessarily haploid males, were used as a standard for ploidy analysis. Smaller peaks of diploid or tetraploid cells can be recognized as well.
